# RNA regulators responding to ribosomal protein S15 are frequent in sequence space

**DOI:** 10.1093/nar/gkw754

**Published:** 2016-08-31

**Authors:** Betty L. Slinger, Michelle M. Meyer

**Affiliations:** Biology Department, Boston College, Chestnut Hill, MA 02467, USA

## Abstract

There are several natural examples of distinct RNA structures that interact with the same ligand to regulate the expression of homologous genes in different organisms. One essential question regarding this phenomenon is whether such RNA regulators are the result of convergent or divergent evolution. Are the RNAs derived from some common ancestor and diverged to the point where we cannot identify the similarity, or have multiple solutions to the same biological problem arisen independently? A key variable in assessing these alternatives is how frequently such regulators arise within sequence space. Ribosomal protein S15 is autogenously regulated via an RNA regulator in many bacterial species; four apparently distinct regulators have been functionally validated in different bacterial phyla. Here, we explore how frequently such regulators arise within a partially randomized sequence population. We find many RNAs that interact specifically with ribosomal protein S15 from *Geobacillus kaustophilus* with biologically relevant dissociation constants. Furthermore, of the six sequences we characterize, four show regulatory activity in an *Escherichia coli* reporter assay. Subsequent footprinting and mutagenesis analysis indicates that protein binding proximal to regulatory features such as the Shine–Dalgarno sequence is sufficient to enable regulation, suggesting that regulation in response to S15 is relatively easily acquired.

## INTRODUCTION

Interactions of an mRNA with its environment are frequently used to modulate gene expression in response to a wide variety of cues. In bacteria there are a plethora of *cis*-regulatory elements found in mRNA 5′ untranslated regions (5′-UTRs). These regulators typically consist of a structured sensor (aptamer) that upon ligand binding alters the conformation of the mRNA to mask or reveal regulatory elements such as ribosome-binding sites, intrinsic transcription terminators or nuclease recognition sites (1). Such RNA regulators interact with a growing number of signals including metal ions (2), small molecules (3), proteins (4) and temperature (5), to control metabolic pathways (6), as well as stress and virulence responses (7–9). Furthermore, such RNA sensors often have complex tertiary structures that have exquisite specificity for their ligands (10).

Many bacterial RNA regulators are distributed to very narrow groups of organisms, despite their key roles in regulating cellular metabolism and complex tertiary structures (11,12). Nearly 40% of families of homologous RNA structures are only identified in species that share the same genus or family (13). This narrow distribution leaves many cases in which the same biological process may be regulated by apparently non-homologous RNA regulators. For example, at least three structurally distinct RNAs interact with S-adenosyl methionine to regulate genes in methionine metabolism in different bacterial species (14–17). Furthermore, for one of the SAM-binding RNA structures there are several distinct variations that based on sequence and secondary structure alone are not obvious homologs; only tertiary structure analysis revealed their evolutionary relationship (18). This situation is far from unique, two RNAs are known to interact with the second messenger cyclic-di-GMP (7,19), and three RNAs interact with pre-queosine_1_ (preQ_1_), a metabolic precursor to the tRNA modification queosine (20–22). This phenomenon is not limited to RNA regulators that interact with small molecules. Structured RNA elements responsible for autogenous regulation of ribosomal protein operons display similar levels of diversity. Homologs of ribosomal protein L20 interact with three different mRNA regulatory structures (two in *Escherichia coli* and one in *B. subtilis*) in addition to the rRNA binding site, and ribosomal protein S15 interacts with at least four distinct mRNA structures in different bacterial phyla (23–26).

Understanding the factors that drive the diversity of RNA regulators observed is a key question facing the RNA community. Are structured RNA regulators responding to the same ligand truly non-homologous, or are they simply too distinct for their similarities to be recognized? The answers to these questions will inform choices of ncRNA drug targets and strategies to prevent resistance (27,28), as well as shed light on whether the considerable body of work using RNA as computational model of evolution is directly applicable to RNAs observed in nature (29). However, the difficulty associated with identifying distantly related homologs makes addressing such questions using phylogenetic and comparative genomic approaches alone challenging. In addition, due to the laborious nature of ncRNA discovery, it is likely that many ncRNAs have not yet been identified, thus there is almost certainly missing data in studies of natural ncRNA regulators.

Understanding the evolutionary processes that lead to the evolution of RNA regulators also hinges upon understanding how frequently such regulators may appear in within sequence space. Essentially, knowing the expectation value for generating a regulator from random sequence. The same RNA structure tertiary structure may be supported by diverse primary sequences and secondary structure arrangements, and there may be many different tertiary structure solutions to the same biological problem. However, it is also possible that few distinct solutions exist. *In vitro* selection has been used to identify RNA aptamers that interact with a variety of ligands. From these experiments it is clear that creation of high-affinity RNA-ligand interactions is relatively facile, and that *in vitro* selected RNAs have many of the same sequence features as natural structured RNAs (30). However, such experiments often identify relatively simple structures compared with many of those identified in nature, largely due to the increased prevalence of shorter motifs in limited sequence pools (31). In addition, the conversion of *in vitro* derived aptamers into gene regulators is often non-trivial (32). Yet, such experiments are a way to examine the biophysically possible solutions without the biases inherent in natural sequences.

In order to better understand the factors contributing to the diversity of S15 interacting mRNA structures observed across different bacterial phyla, we used SELEX to explore the potential RNA-binding pool for *Geobacilus kaustophilus* ribosomal protein S15 (33,34). In addition to its primary function within the ribosome, in different bacterial species ribosomal protein S15 interacts with at least four different mRNA regulators to control its own translation (23,25,26,35). Our previous work and that of others indicated that *G. kaustophilus* S15 (Gk-S15) does not interact with the mRNA regulator originating from *E. coli* (Ec-mRNA) (36,37), and the thermophillic nature of *G. kaustophilus* makes the protein easy to handle and purify. Therefore, we used the *E. coli* mRNA regulatory sequence as the basis for a randomized RNA pool and conducted *in vitro* selection to identify sequences that interact with Gk-S15. From our final sequence pool we cloned six distinct aptamers. All were able to bind Gk-S15 with varying affinities in the nM range. Using an *E. coli* host and a *lacZ* reporter system, we demonstrate that two-thirds of these aptamers enable Gk-S15-responsive gene regulation. To further examine what factors are important for conferring gene regulatory ability, we characterized the protein-binding site of several sequences using footprinting assays and mutagenesis to find that RNAs allowing regulation have protein-binding sites in close proximity to regulatory regions compared to RNAs that do not regulate. From this work, we conclude that RNA regulators responding to S15 are common within sequence space. Thus, the diversity of S15 regulatory RNAs is driven not only by co-evolution with the protein partners (37), but also by the relatively high frequency of such regulators appearing in sequence space.

## MATERIALS AND METHODS

Protein Overexpression and Purification: The *rpsO* open reading frame encoding S15 was polymerase chain reaction (PCR) amplified using whole genomic DNA and cloned into pET-HT overexpression vector similarly to previously described (26). Sequence verified plasmid was transformed into chemically competent BL-21 cells (DE3). Protein expression and purification for each S15 homolog was conducted as described previously (26).

*In vitro* selection: RNA selection experiments proceeded as described (38) using the template 5′- TAATACGACTCACTATAGGTGCGTAACGTACACT-N_30_- TCATTCTATATACTTTGGAGTTTTAAAATGTCTCTAAGTACTGAAGCAACAGCT where N_30_ represents 30 random nucleotides, and the T7 RNA polymerase promoter sequence is underlined. Transcription reactions were performed using T7 polymerase (39), then purified by 6% denaturing PAGE. Bands were visualized using UV shadow, excised and the RNA eluted (in 200 mM NaCl, 1 mM EDTA ph 8, 10 mM Tris-HCl pH 7.5) and ethanol precipitated.

In each round of selection 300 pmol of RNA were renatured at 42°C for 15 min, and then filtered through 0.45 μM nitrocellulose to remove any nitrocellulose binders. RNAs in the flow-through were incubated with Gk-S15 in Binding Buffer A (50 mM Tris/Acetate, pH 7.5, 20 mM Mg-Acetate, 270 mM KCl, 5 mM dithiothreitol, 0.02% bovine serum albumin) at 25°C for 30 min, then RNA-Gk-S15 complexes were isolated by filtering with nitrocellulose. After two washes the bound RNAs were eluted from the filter (7 M Urea, 100 mM Na_3_C_6_H_5_O_7_, 3 mM EDTA pH 8.0) and purified using isopropanol. The RNA aptamers were reverse transcribed using M-MuLV, and cDNA amplified using mutagenic PCR (40). This pool was then used to transcribe RNA for the next round of selection (Supplementary Figures S1 and S2). After 11 rounds, cDNA was ligated into the pCR 2.1 vector (making pCR–RNA) to examine individual sequences.

Binding and Competition Assay: For natural sequences, DNA corresponding to the 5′-UTR of the *rpsO* gene was PCR amplified using species-specific primers with the T7 promoter sequence added within the forward primer sequence (Supplementary Figure S3). Genomic DNA extracted from the species was used as template. For all synthetic sequences the pCR–RNA was used as template to amplify DNA. T7 RNA polymerase was used to transcribe RNA and transcription reactions were purified and eluted as described in SELEX experiment (39). Purified RNA (10 pmol) was 5′-labeled with ^32^P-ATP and purified as previously described (41). Binding assays were performed and quantitated as previously described using nitrocellulose and nylon membranes (GE Healthcare) (26). Dissociation constants (K_D_) and maximum fraction bound (F_max_) were calculated by fitting the fraction of protein bound RNA (fraction bound, Fb) at a range of protein concentrations to the equation Fb = (F_max_*[S15])/([S15)+KD) where [S15] is the protein concentration using Excel Solver to minimize the residuals. Mutations to the mRNAs were constructed by site-directed mutagenesis (Supplementary Figure S3).

Footprinting assays: The RNA–protein binding reaction described above was used for RNAse probing assays. After incubation, 1 μl RNAse A (1 μg/ml, Ambion) or VI (1:400 dilution of 0.1 U/μl, Ambion) was added and the reaction incubated for 15 min at 25°C. The nuclease was inactivated with inactivation/precipitation buffer (Life Sciences) and RNA fragments recovered by ethanol precipitation. Precipitated RNAs were suspended in 10 μl Urea Loading solution (Life Sciences) and incubated 5 min 95°C. Partial hydroxyl cleavage reactions were generated by incubating RNA in 50 mM Na_2_CO_3_ pH 9.0, 1 mM EDTA at 95°C for 7 min. Denaturing T1 reaction (1:10 dilution) was conducted according to manufactures protocol (Ambion). For in-line probing, 5′-labeled RNA was incubated 40 h at 25°C in reaction buffer (20 mM MgCl_2_, 100 mM KCl, 50 mM tris pH 8.3). The reaction was stopped using Urea loading solution (10 M Urea, 1.5 mM EDTA). For lead(II)-probing, protein binding reactions were incubated for 10 min with 15 uM Pb(II)-acetate (300 mM stock prepared directly before use). Reactions were stopped with the addition of EDTA (final concentration 25 μM), and the addition of Urea loading solution. Reactions were loaded on 10% denaturing Acrylamide/Bis-acrylamide gel to separate RNA fragments. The gel was dried and visualized using a GE Healthcare STORM 820 phosphorimager and ImageQuant software.

Plasmid Construction: All synthetic sequences were cloned into the pBS3-RNA plasmid as a translational fusion with *lacZ* using primers containing EcoRI and SalI restriction sites and template from TOPO PCR 2.1 cloned PCR product (Supplementary Figure S3). The *lacZ* sequence requires a start codon from the fused *rpsO* sequence. All enzymes for molecular biology were purchased from New England Biolabs unless otherwise noted.

pS15 protein expression plasmids were constructed by amplifying the open reading frame from genomic DNA with a forward primer containing SacI site and a strong ribosome-binding site corresponding to the *E. coli* S15 ribosome binding site and an 8 nucleotide linker (Supplementary Figure S3) preceding the ATG start site and subsequent codons. The reverse primer contained an XbaI site. After digestion, the PCR product was cloned into the pBAD33 vector (ATCC 87402) digested with the same enzymes. All pS15 were sequence verified. S15 mutants were constructed using site-directed mutagenesis.

*LacZ* regulatory assays: As described previously (37), K12: Δ*rpsO E. coli* cells were co-transformed with pRNA and pS15 plasmid (made competent using the Z-competent buffer system, Zymo Research). A single colony was used to start overnight cultures, grown ± L-arabinose (15 mM) at 37°C, 225 rpm, then diluted the next day to OD600 = 0.15 in fresh media (LB + 100 µg/ml ampicillin + 34 µg/ml chloramphenicol ± 15 mM L-arabinose). At stationary phase (5 h after dilution) 1 mM IPTG was added to induce β-galactosidase expression. After 30 min, 100 µg/ml spectinomycin was used to stop initiation of protein translation, and the cultures assayed immediately according to Miller to determine the levels of reporter expression (42). Fold repression = (Miller units of – L-arabinose)/(Miller units of + L-arabinose). All RNA/S15 combinations were examined with 3+ independent replicates. Regulation was considered biologically significant if greater than 2.5-fold repression was observed, and the fold repression was significantly different (*P* < 0.05) than that observed with an empty pBAD33 vector.

## RESULTS

### *In vitro* selection of RNA aptamers for Gk-S15

We carried out 11 rounds of SELEX on a randomized RNA pool to isolate RNAs that bind S15 from *G. kaustophilus* (Gk-S15) with a high affinity (Supplementary Figure S1A). Because there is no regulatory interaction between the mRNA regulator from *E. coli* (Ec-mRNA) and Gk-S15 (37), and the Ec-mRNA allows robust expression in *E. coli*, the randomized RNA pool was based upon Ec-mRNA sequence: 5′-TGCGTAACGTACACT-N_30_- TCATTCTATATACTTT**GGAG**TTTTAAA**ATG**TCTCTAAGTACTGAAGCAACAGCT, where primer binding regions are underlined, and N_30_ denotes a randomized region of 30 nucleotides (Supplementary Figure S1B). The native *E. coli rpsO* start codon is retained in the 3′ primer region (bolded), and the native Shine-Dalgarno sequence is not within the randomized region (bolded). Protein-binding RNAs were selected using nitrocellulose filter binding. Mutagenic PCR was used at each round to amplify cDNA as well as increase sequence diversity of the non-primer regions.

**Figure 1.**
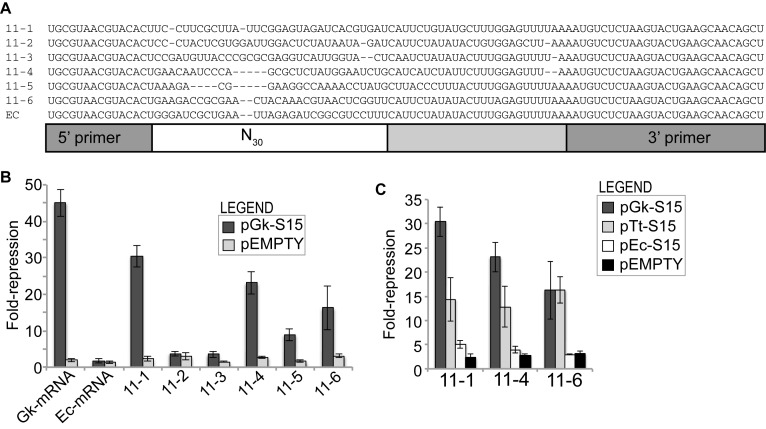
Selected RNAs. (**A**) Sequence alignment of selected sequences indicating the 5′ and 3′ primer regions and the randomized region. A version of this alignment highlighting differences between the sequences in color can be found in Supplementary Figure S4. (**B**). Selected RNAs regulate gene expression in response to S15. Miller assays were performed to assess the regulatory capacity for a selected RNA in response to the S15 homolog indicated, or a ‘no S15’ plasmid (pEMPTY). The six individual sequences isolated from the round 11 sequence pool were assessed for *in vivo* response to *G. kaustophilus* S15 (pGk-S15). (**C**) The regulatory response of RNAs 11–1, 11–4 and 11–6 was assessed in response to S15 homologs from *T. thermophilus* (pTt-S15) and *E. coli* (pEc-S15). Data corresponding to pGk-S15 and pEMPTY interactions are re-plotted from Figure 1B for comparison. All error bars correspond to standard error for 3 or more independent replicates.

During eleven rounds of selection we decreased the concentration of Gk-S15 (from 1250 nM to 100 nM) and the population binding affinity dramatically increased from a K_D_ of >1 μM in the unselected population to 150 nM at the final round (Supplementary Figure S2A and B). To assess the affinity of individual sequences in the Round 11 pool, we isolated and sequenced six individuals from this population. Each sequence was unique and the sequences are diverse from one another, containing no common sequence or motif in the randomized region (Figure 1A, Supplementary Figure S4). Additionally, these sequences are predicted to fold into unique secondary structures using RNAfold of the Vienna RNA Package (Supplementary Figure S4) (43). These six RNAs represent a small selection of potential unique S15 binders, as we demonstrate via subsequent next generation sequencing analysis of the SELEX experiment (data to be published elsewhere). Nitrocellulose filter binding assays were performed using Gk-S15 and 5′-end labeled RNA for each of the six sequences (Table 1, Supplementary Figure S5). All the RNAs were able to bind Gk-S15, although the range of binding affinities spans several orders of magnitude, and the maximum fraction of RNA bound by protein (F_max_) also varies between 20% and 80%. We identified one sequence, 11–1, which has a binding affinity approximating that of the native mRNA interaction for Gk-S15 (Gk-mRNA, ∼1 nM, F_max_ = 70%). Four of the remaining sequences still strongly bind Gk-S15 (K_D_ = 8.5–20.7 nM), although 11–4 displays a low F_max_ of only ∼20%. The final sequence, 11–6, has a relatively weak binding affinity (289 nM). These results suggest that our final sequence pool is likely very diverse and indicate that our SELEX experiment was successful in selecting RNA that interact with Gk-S15.

**Table 1.** Dissociation constants for selected RNA sequences with S15 from *Geobacillus kaustophilus* (Gk-S15)

RNA	K_D_ (nM)	Standard error	F_MAX_ (%)	Standard error
Gk-mRNA	0.7	0.02	66	5.7E-03
Ec-mRNA	>>100	n/a	32	1.0E-02
11–1	0.9	0.02	70	4.9E-02
11–2	9.7	1.6	79	3.8E-03
11–3	20.7	15.2	40	4.3E-02
11–4	8.5	2.0	21	1.2E-02
11–5	10	1.4	44	1.6E-02
11–6	289	121	55	4.0E-02

### RNAs regulate gene expression in response to Gk-S15

To assess whether any of the selected aptamers allowed regulation, we used an *in vivo* regulation assay to screen the six cloned sequences. Our regulatory assay consists of a two-plasmid system that assesses whether over-expressed Gk-S15 interacts with an RNA to regulate β-galactosidase expression in the cell. One plasmid contains an RNA-*lacZ* reporter with a selected RNA sequence cloned upstream and in-frame with *lacZ* and under the control of an IPTG-inducible pLac promoter. A second plasmid carries the *G. kaustophilus rpsO* coding sequence under the control of an L-arabinose-inducible promoter (37). The plasmids are co-transformed into an *E. coli* K12:Δ*rpsO* strain (44). The regulatory assay is performed with cultures grown in the absence and presence of L-arabinose to induce S15 expression. At stationary phase (OD_600_ ≈ 1.5) we performed a 30-min mRNA-*lacZ* induction and measured the change in gene expression between + and – arabinose conditions. During log-phase growth, over-expressed S15 is rapidly incorporated into the ribosome.

Strikingly, four of the six cloned sequences enabled a range of regulatory responses to Gk-S15, while two showed no ability to regulate gene expression (Figure 1B). All four regulators had significantly higher β-galactosidase activity in the absence of L-arabinose, indicating that they behave as genetic ‘OFF’ switches similarly to the natural regulators. The maximal amount of reporter expression allowed by each aptamer differed (∼1000–5000 Miller Units, Supplementary Figure S6A and B), impacting the fold-repression for each sequence. We typically observe 1.5- to 3-fold-repression in the absence of over-expressed protein (pEMPTY), likely due to the metabolic effects of the added sugar. The strongest binder, 11–1, enabled the strongest gene regulatory response, 30.4-fold-repression, approximately 24-fold higher than the pEMPTY control. Two of them, 11–4 and 11–5, have modest binding affinities, yet both regulate reporter expression in response to Gk-S15 (23.1- and 8.9-fold-repression, ∼8.5- and 5-fold higher than the pEMPTY control, respectively). Finally, 11–6 has the weakest binding affinity of the six individuals, yet still shows a regulatory response to Gk-S15 (16.3-fold-repression, 5-fold higher than the pEMPTY control). Thus, *in vitro* binding affinity and maximum fraction of RNA bound in vitro (F_max_) do not correlate with regulatory capability. The 11–2 and 11–3 bind Gk-S15 strongly *in vitro*, yet neither was able to regulate gene expression *in vivo*. While all of the regulators show weaker regulatory response than the native Gk-S15:mRNA interaction (Figure 1B), the fold-repression observed for the selected RNA regulators in the range observed for the native *E. coli* S15:mRNA interaction (∼16-fold repression) (37).

### RNA regulators likely share some tertiary structure characteristics

Our previous work indicates that different S15 homologs recognize RNA regulatory sequences using different motifs. The rRNA binding site for S15 is bipartite and consists of both a three-helix junction and a GU/GC motif one helical turn distal from the junction. S15 homologs from *G. kaustophilus* and *Thermus thermophilus* both require a mimic of the 3 helix junction (3HJ) in order to enable binding to regulatory mRNA structures, whereas the homolog from *E. coli* requires a GU/GC motif and a bulged adenosine that is not an obvious mimic of the 3HJ (45,46). By assessing whether different S15 homologs are able to interact with the regulatory RNAs we expect to gain information about the potential protein-binding motifs within the RNAs. We examined whether our three most promising regulatory RNAs, 11–1, 11–4 and 11–5, respond to homologs of S15 from *T. thermophilus* and *E. coli*. Each of these RNAs regulate in response to not only Gk-15, but also the S15 homolog from *T. thermophilus* (Tt-S15) (Figure 1C, Supplementary Figure S6C). The RNAs did not respond strongly to the *E. coli* S15 homolog (Ec-S15). This result is surprising because the original RNA pool was based on Ec-mRNA. These results suggest that the selected RNAs likely contain tertiary structures that mimic the 3HJ rather than the more easily identifiable GU/GC motif.

### Selected sequences interact with the rRNA-binding face of Gk-S15

**Figure 2.**
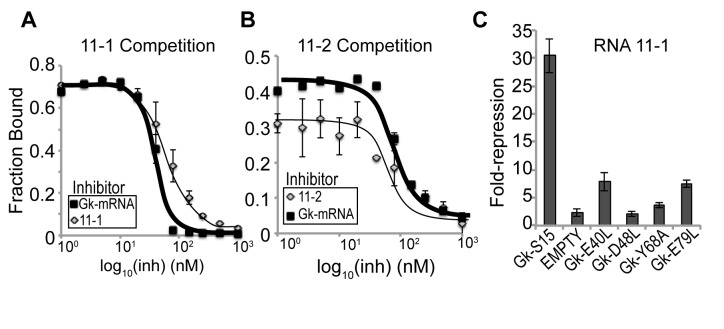
RNAs 11–1 and 11–2 interact with the same face of Gk-S15 as Gk-mRNA. *In vitro* competition binding curves and *in vivo* regulation data to assess how synthetic RNAs 11–1 and 11–2 interact with Gk-S15. (**A**) Titration of non-labeled competitor, ‘Inhibitor’ (Gk-mRNA or 11–1), with ^32^P-labeled 11–1 and Gk-S15. (**B**) Titration of non-labeled competitor, ‘Inhibitor’ (Gk-mRNA or 11–2), with ^32^P-labeled 11–2 and Gk-S15. (**C**) *In vivo* regulation assay for RNA 11–1 with pGk-S15, pEMPTY, mutations to Gk-S15 binding face (D48L and Y68A) or mutations to non-binding face of Gk-S15 (E40L, E79L). Data corresponding to pGk-S15 and pEMPTY are re-plotted from Figure 1B for comparison. All error bars correspond to standard error for 3 or more independent replicates.

To better understand why some RNA sequences enable Gk-S15-based gene regulation, whereas others do not, we closely examined the two RNAs with the lowest dissociation constants, one of which is also a regulator (11–1) and the other of which is not (11–2). Previous studies have shown that the naturally occurring RNA regulators of the *rpsO* operon interact with a conserved set of amino acids in the S15 protein, all of which fall on the same protein face (26,36,47). We performed several experiments to assess whether these RNAs interact with the same face of Gk-S15 as the Gk-mRNA. First, an *in vitro* competition experiment was performed using a fixed amount of 5′-end labeled RNA, Gk-S15 and an increasing amount of non-labeled competitor RNA. We find that both 11–1 and 11–2 are displaced by Gk-mRNA, and each other, from Gk-S15 (Figure 2A and B). This suggests that both RNAs bind the same face of Gk-S15. Second, nitrocellulose binding assays were performed with both 11–1 and 11–2 RNAs and the S15 homologs from *T. thermophilus* (Tt-S15) and *E. coli* (Ec-S15). We find that both RNAs are only able to bind Tt-S15, and not Ec-S15 (Table 2, Supplementary Figure S7A and B). This is consistent with our previous result that 11–1 regulates in response to Tt-S15 but not Ec-S15, and further indicates that the rRNA binding face is likely used to interact with the selected RNAs, although the features for binding rRNA, mRNA and selected RNAs may be different.

**Table 2.** Binding constants between 11–1, 11–2 and S15 homologs from *Thermus thermophilus* (Tt-S15) and *E. coli* (Ec-S15)

RNA	Protein	K_D_ (nM)	Standard error	F_MAX_ (%)	Standard error
11–1	Tt-S15	94.9	22.5	78	3.7E-03
11–1	Ec-S15	>300	n/a	n/a	n/a
11–2	Tt-S15	39	6.27	25	6.5E-00
11–2	Ec-S15	>300	n/a	n/a	n/a

We further examined the 11–1 RNA–protein recognition in our regulatory assay using several Gk-S15 mutants (36). We were unable to examine 11–2 in this manner because it is not a functional regulatory RNA. Mutations to the binding face of Gk-S15 (Y68A and D48L) prevented RNA recognition and subsequent gene regulation (Figure 2C, Supplementary Figure S6D). These individual amino acids were found to be essential for autoregulation of the native Gk-S15 regulatory Gk-mRNA (36). However, mutations to the non-binding face of Gk-S15 (E40L and E79L) do not prevent Gk-S15 from regulating gene expression in response to 11–1. Taken together, these data show 11-1 not only binds on the same face of Gk-S15 as its native RNA regulator, but likely also utilizes similar amino acids for recognition.

### Gk-S15 binds 11–1 near the Shine–Dalgarno sequence

**Figure 3.**
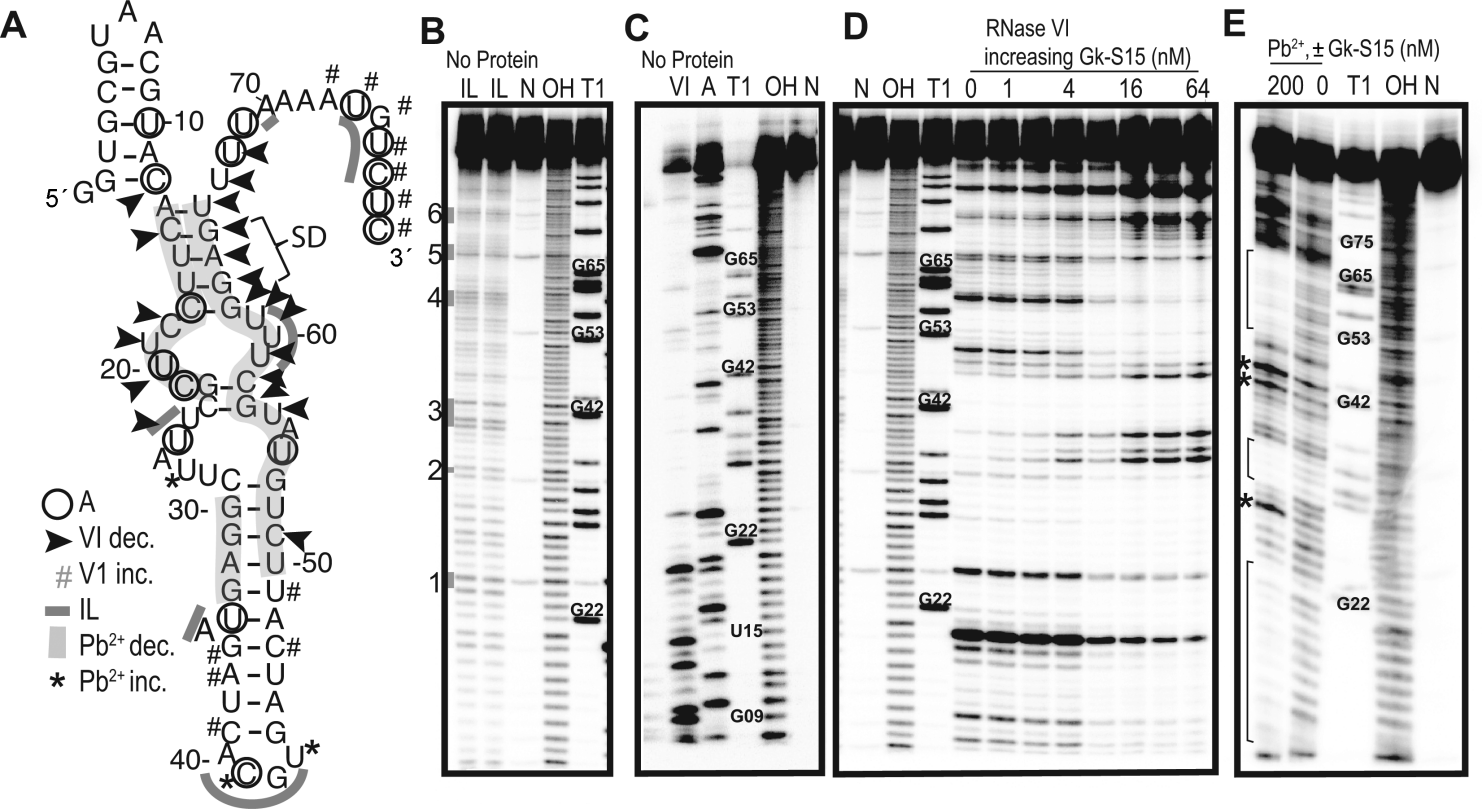
Structure probing elucidates secondary structure of RNA 11–1. For all individual gels, no reaction (N), hydroxyl cleavage (OH) and denaturing RNase T1 (T1) are indicated. All cleavage products have been separated by denaturing 10% PAGE. (**A**) Predicted RNA 11–1 structure with all footprinting data mapped to the structure. (**B**) Two independent replicates of in-line probing reactions (IL). (**C**) RNase VI (V1), RNase A (A) in the absence of Gk-S15. (**D**) Titration of Gk-S15 with RNase VI, where protein concentration (nM) is indicated. (**E**) Lead(II)-probing reactions (Pb^2+^) in the presence and absence of 200 nM Gk-S15.

All of our sequences retain the native Shine–Dalgarno sequence and start codon, yet only some of them regulate gene expression. While RNA-S15 binding must occur to regulate gene expression, it is not necessarily sufficient. To further investigate this, we examined the binding interaction between Gk-S15 and the best performing regulatory RNA, 11–1 to more clearly establish how Gk-S15 recognizes the RNA to enable regulation. RNA footprinting experiments were performed to elucidate the secondary structure features in 11–1 that may be essential for regulation in response to Gk-S15 (Figure 3A–E). Using 5′-labeled 11–1 in the presence and absence of Gk-S15, RNA secondary structure was probed using RNase VI (VI-, cleaves double-stranded regions and is not base-specific), RNase A (A-, cleaves single-stranded cytosines and uracils), in-line probing (IL-, cleaves flexible and likely single-stranded regions and is not base-specific) and lead(II) probing (Pb-, cleaves flexible regions and is not base-specific).

Using this structure probing data, we predicted a structure for 11–1 when bound to Gk-S15 (Figure 3A). Overall, 11–1 appears relatively unstructured in the absence of protein. This is especially apparent with the number of in-line probing and lead(II)-cleavage products in the absence of protein (Figure 3B, E). Upon Gk-S15 binding, the RNA takes a more defined secondary structure. The intensity of VI-cleavage products corresponding with U10, C12, U19, U20 and U25 decreases in the protein-bound RNA, suggesting this region is shielded from RNase cleavage by Gk-S15, or is becoming single-stranded (Figure 3D). Additionally, in the protein-bound RNA, there is reduced lead(II)-cleavage in nucleotides C13 through G22, as well as in G30 through G32, suggesting this region is not flexible when protein is bound (Figure 3E). The RNA sequence spanning nucleotide U49 through U67 is also likely to be involved in Gk-S15 recognition. In the presence of Gk-S15, there is reduced lead(II)-cleavage in the region from U52 through G65 (Figure 3E). Also, the VI-cleavage product intensity for U50 through U52 decreases as Gk-S15 concentration is increased (Figure 3D). There is strong VI-protection of nucleotides U60, U61 and U68, and general shielding of the remaining nucleotides 62 through 67, suggesting Gk-S15 binding and shielding of this region. All of these results suggest Gk-S15 binds this portion of the RNA. The native Shine–Dalgarno sequence within this RNA corresponds to nucleotides 62–65. Thus our results indicate that regulation may be largely due to occlusion of the Shine–Dalgarno sequence, either by GK-S15 directly or through stabilization of secondary structure in this region.

The footprinting data suggest that the central part of the RNA sequence, G36 through C47, folds into a hairpin. As Gk-S15 concentration is increased, there is increased VI-cleavage product intensity at nucleotides G36, A37, C39, C47, indicating that Gk-S15 does not protect this region, and it is double stranded. Also, in the presence of Gk-S15, the lead(II)-cleavage of the 11–1 RNA increases for nucleotides C41 and U43; there are IL-cleavage products for A40 through U43 (Figure 3B) as well as RNase A-cleavage for C41 (Figure 3C). This all suggests the formation of a stem loop region. Taken together, nucleotides G36 through C47 likely fold into a hairpin that does not directly interact with Gk-S15 upon protein binding.

### Mutagenesis experiments confirm Gk-S15 binding regions of 11–1

**Figure 4.**
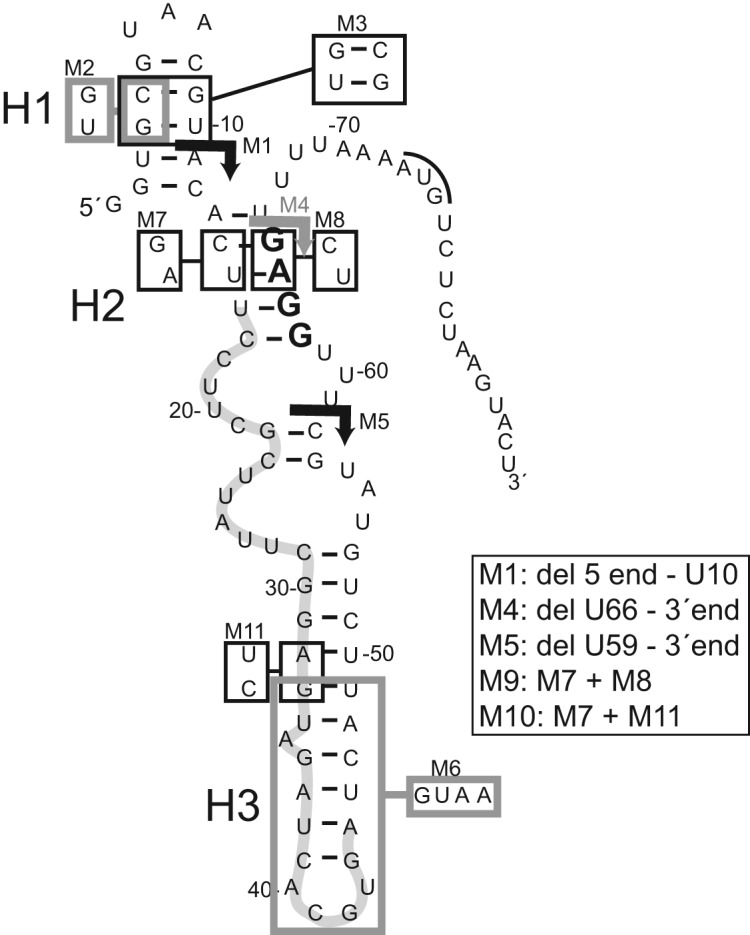
*In vitro* binding assays and mutagenesis confirm predicted RNA 11–1 structure. *In vitro* binding assays were performed with Gk-S15 and mutant versions of RNA 11–1. These results largely confirm the footprinting results. Truncation sites and specific mutations to 11–1 are shown. The Shine–Dalgarno sequence is in bold, a bar is placed over the AUG start codon and putative helices H1, H2 and H3 are indicated. The resulting N_30_ region in RNA 11–1 is highlighted in grey, U16-A45.

To confirm our secondary structure model, a variety of mutations to the 11–1 RNA sequence were designed and the ability for these to bind Gk-S15 was tested using filter-binding assays (Table 3, Figure 4, Supplementary Figures S8 and S9). Mutations to the 5′-region of the aptamer were first assessed. The first 5′ truncation of 11 nucleotides (11–1-M1) completely abolishes Gk-S15 binding, suggesting that this region is critical for the RNA–protein interaction. When constraints derived from our footprinting data are utilized, the RNA structure prediction program RNAfold (43) suggests this region of the RNA folds into a small hairpin. To establish whether this structure forms, we created a mutation to this region that prevents the putative double helix formation (11–1-M2). This mutation abolished Gk-S15 recognition, and the compensatory mutation (11-1-M3) successfully restored Gk-S15 binding. Together these results suggest nucleotides U1 through A11 fold into a hairpin whose presence is required for Gk-S15 binding.

**Table 3.** Binding constants of RNA 11–1 Mutants with Gk-S15

RNA	K_D_ (nM)	Standard error	F_MAX_	Standard error
11–1	0.9	0.02	0.70	4.9E-02
11–1-M1	>100	n/a	n/a	n/a
11–1-M2	56.5	5.74	0.56	2.0E-02
11–1-M3	20.9	6.3	0.85	3.7E-03
11–1-M4	7.7	5.89	0.36	5.8E-03
11–1-M5	>100	n/a	n/a	n/a
11–1-M6	4.63	0.8	0.69	2.3E-02
11–1-M7	>300	n/a	n/a	n/a
11–1-M8	84.5	14.5	0.71	1.4E-02
11–1-M9	>300	n/a	n/a	n/a
11–1-M10	>300	n/a	n/a	n/a

Truncations to the 3′ end of the RNA sequence confirm that many of these nucleotides are not required for binding Gk-S15 (Table 3, Figure 4). A 22 nucleotide 3′-truncation (11–1-M4) only slightly affected protein binding, but a 29 nucleotide 3′-truncation (11–1-M5) abolished binding. These results suggest that Gk-S15 does not require the 3′ nucleotides deleted in M4 to bind the RNA. This finding supports our footprinting assays, which indicate this region remains unstructured even in the presence of protein. We also confirmed that the putative stem loop region spanning G33 through U49 suggested by the lead(II)- and VI-probing data are not required for binding Gk-S15. Replacement of this entire region with a GUAA tetraloop (11–1-M6) did not affect recognition by the protein.

Mutations to the central core of the putative RNA 11–1 structure drastically affect Gk-S15 binding (Table 3, Figure 4). Based on the protection from nuclease cleavage observed in the footprinting assays, we created a mutation to helix 2 (11–1-M7) that abolished Gk-S15 binding. When we mutated the opposite side of the helix, 11–1-M8, the dissociation constant changed significantly, but protein binding still occurred. The compensatory mutant did not compensate for the RNA secondary structure, as no Gk-S15 binding was apparent with 11–1-M9. Testing alternative base-pairing partners for the nucleotides C14-U15, 11–1-M10, also did not compensate the 11–1-M7 defect (see Supplementary Figure S10 for alternative structure diagram, and Supplementary Figure S11 for footprinting data mapped to this alternative structure). Therefore, we suspect that 11–1-M7 mutated a nucleotide-specific interaction for Gk-S15. Based on these results and the footprinting data, we believe we have the correct structure of RNA 11-1 upon Gk-S15 binding.

### Gk-S15 binds 11–2 at regions distal to potential regulatory features

**Figure 5.**
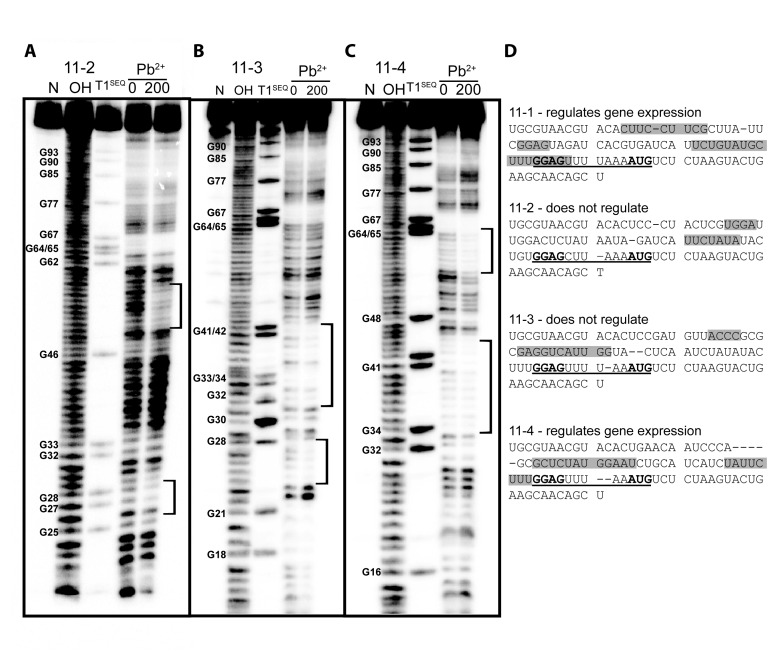
Lead(II)-probing shows that the protein binding site on the selected RNA is critical for determining whether the RNA allows regulation. Lead(II)-probing of sequences (**A**) 11–2, (**B**) 11–3 and (**C**) 11–4 in the presence (200 nM) and absence (0 nM) of Gk-S15. All numbering corresponds to the aligned sequences shown in (**D**). No reaction (N), hydroxyl cleavage (OH) and denaturing RNase T1 (T1^SEQ^) and lead(II) probing (Pb^+2^) lanes are indicated for each RNA. (**D**) Sequences of RNAs 11-1, 11–2, 11–3 and 11–4 showing sites of reduced lead(II)-cleavage upon protein binding (shaded regions). Raw data for 11–1 can be found in Figure 3.

RNA 11–2 was one of two selected RNA aptamers that did not regulate gene expression *in vivo*. To better understand what elements allow this RNA to interact with the protein *in vitro*, but result in no *in vivo* functionality, we performed footprinting assays on this RNA. Again, we used RNase VI, RNase A, RNase T1, lead(II)-probing and in-line probing on 5′-end labeled RNA sequence in the presence and absence of Gk-S15 (Supplementary Figure S12A–C, Figure 5A). Overall, the RNA appears unstructured in the absence of Gk-S15. This is especially evident in the lead(II)-cleavage footprint without Gk-S15 (Figure 5A) and the distinct number of strong IL-cleavage products (U23,C24, U26, U69, U70, common numbering as Figure 5D) (Supplementary Figure S12B).

Protein binding does little to affect the cleavage patterns observed and our data suggest that Gk-S15 does not bind any part of the sequence that may be considered important for regulation. In the lead(II)-footprint we observe reduced cleavage of nucleotides 26 through 29 and of the region 51 through 56 (Figure 5A). This strongly suggests that the protein-bound structure of the RNA forces these regions into a fixed structure. Additionally, there is protection of C53 from VI-cleavage in the presence of the protein (Supplementary Figure S12A), which corroborates the lead(II)-footprint and suggests this region is important for binding Gk-S15. There is no apparent shielding or changes in cleavage patterns to the 3′-region of the RNA where transcription and translational elements are located. More specifically, up to 500 nM Gk-S15 does little to affect the A- and VI-cleavage patterns (Supplementary Figure S12C). To corroborate our putative model for Gk-S15 binding to 11–2 in the C51 region, we designed and tested a mutation to this region of the RNA (11–2-M1, nucleotides 64–66, TAT→GGG) using filter binding. This mutation was sufficient to prevent binding by Gk-S15 (K_D_ > 300, F_MAX_ 0.068 ± 0.044, Supplementary Figure S12D).

### Protein binding proximal to regulatory region is sufficient to allow gene regulation

Based on the 11–1 and 11–2 examples we hypothesize that protein binding to RNA regions proximal to the native regulatory features is sufficient to allow regulation. To provide further evidence for this hypothesis we examined an additional two selected RNAs, 11–3 and 11–4. One of which regulates, the other of which does not (Figure 1). Based on the extensive structural probing and mutagenesis conducted for 11–1 and 11–2, we find that lead(II)-probing provides the most direct information regarding protein binding sites. Therefore we examined 11–3 and 11–4 using lead(II)-probing (Figure 5B and C). RNA 11–3 is shows protection from lead(II)-probing predominately in the 5′- end of the RNA, including nucleotides 24–27 (aligned sequence numbering) and 32–42. No protection is observed in regions of the RNA responsible for gene regulation (nucleotides 64–77). This result is consistent with 11–3's lack of regulatory activity. RNA 11–4 shows significant protection in nucleotides 33–45 and 55–63. This result is consistent with the regulatory activity observed for 11–4. Thus, protein binding to the regulatory region of the RNA is sufficient to allow regulation with Gk-S15 for our *in vitro* selected aptamers (Figure 5D).

## DISCUSSION

Our goal was to use *in vitro* selection to explore how readily regulators responding to *G. kaustophilus* S15 could be recovered from a randomized sequence population. From our selected pool we examined six individual aptamers. Each of the sequences is unique and all of them interact with Gk-S15. To our surprise two thirds of the *in vitro* selected aptamers allowed robust regulation in *E. coli*. Our characterization experiments demonstrate that although the aptamers have no detectable sequence similarities in their variable regions, they all likely interact with the same binding face of Gk-S15, and that they respond to the same set of S15 homologs. This finding suggests that the number of RNAs that may interact with this protein is quite large, and therefore RNA sequences that enable regulation are relatively frequent in sequence space.

One limitation of our study is the fixed sequences at the 5′ and 3′ ends of the sequence and the sequence for nucleotides 48–72 was not completely randomized. Past studies have shown that primers sequences have negligible impact on the results of SELEX (48). However, it is likely that our starting sequence has some impact on our results. Our randomization removes the major secondary structure elements present in the RNA. However, the region including nucleotides 48–72 was only subject to mutagenic PCR. We do observe significant diversity among our sequences in this region, but by necessity all of our RNAs that display regulation bind the RNA partially within this region. Thus, the starting sequence likely has some impact on how frequently we identify regulators. However, the observation that S15 interacts with secondary and tertiary structures rather than specific sequence motifs (23,37,46) suggests that any potential sequence bias alone is not directly responsible for the observed frequency.

The mechanism of action utilized by our selected RNA regulators for regulation is only partially explored here. The natural S15 aptamers use a variety of mechanisms to inhibit translation. In *T. thermophilus*, S15 uses a ‘displacement’ mechanism where S15 directly competes for binding the mRNA transcript with the ribosome (25). In *E. coli*, S15 uses an ‘entrapment’ mechanism to regulate expression of the *rpsO* operon in which both Ec-S15 and the ribosome pre-initiation complex bind the mRNA simultaneously. This interaction ultimately prevents 70S assembly and halts translation (46). Entrapment mechanisms are impossible to effectively select for *in vitro*, but biophysical modeling has shown entrapment may allow lower affinity interactions to still regulate efficiently (49). Our results suggest that aptamers resulting from selection of RNA pools including regions proximal to regulatory features, such as the Shine–Dalgarno sequence, start codon and protein-coding nucleotides are likely to have regulatory function as protein binding to these regions is be sufficient to allow regulation. All of our selected regulators behave as genetic OFF switches, and our footprinting and site-directed mutagenesis experiments show that regulatory RNAs 11–1 and 11–4 interact with the protein to occlude the SD sequence, whereas protein binding to non-regulatory RNAs 11–2 and 11–3 does not appear to affect regulatory features. We found no correlation between strength of interaction and regulatory ability. RNAs with modest binding affinity (such as 11–6) can regulate gene expression. This finding is consistent with our previous studies that indicate a low binding affinity may still allow robust regulation (26,37). Based on these data, and the frequency with which regulators were obtained, we believe that Gk-S15 is likely preventing translation using a displacement type mechanism with our selected sequences.

The S15:mRNA regulatory interaction can take different forms, and many naturally-occurring regulatory RNA structures have been described that all perform analogous regulatory functions in different bacterial species (23,25,26,35). Therefore, it is not surprising that we were able to isolate several novel structures that also regulate gene expression in response to S15. However, our data suggest that tertiary structure motifs, which are difficult to discern from primary and secondary structure alone, are important for S15 binding. The natural RNAs all share some identifiable partial mimicry with the rRNA-binding site for S15 at either the three-helix junction or GU/GC motif, and Gk-S15 in particular requires the 3HJ motif within an RNA for regulation (37). Our experiments with alternative S15 homologs suggest that all of the regulatory RNAs likely contain some mimic of this junction since they interact with both Gk-S15 and Tt-S15, which directly recognize this motif, and not Ec-S15, which relies more upon the GU/GC motif.

One of the observations driving this work is the identification of several natural RNAs interacting with the same small-molecule ligand, or with homologous proteins. Our results suggest that ribosomal protein S15 responsive regulators are likely to be independently derived. We find that solutions to this biological problem are frequent in the sequence space we explored. Furthermore, similar to the natural solutions, our selected regulators display specificity for some of the proteins over others. Despite starting with a sequence population derived from the native *E. coli* mRNA, none of our selected regulators interacts with Ec-S15, indicating that selection experiments with different S15 proteins will yield different pools of sequences.

## Supplementary Material

SUPPLEMENTARY DATA
