# Supplementary material for: RNA regulators responding to ribosomal protein S15 are frequent in sequence space
Source: Nucleic Acids Res. 2016 Aug 31;44(19):9331–41. doi: 10.1093/nar/gkw754 (PMC5100602; doi:10.1093/nar/gkw754)
Supplement: SUPPLEMENTARY DATA [file supp_44_19_9331__index.html]

RNA regulators responding to ribosomal protein S15 are frequent in sequence space — RNA regulators responding to ribosomal protein S15 are frequent in sequence space — SUPPLEMENTARY DATA 

# RNA regulators responding to ribosomal protein S15 are frequent in sequence space

## SUPPLEMENTARY DATA

- SUPPLEMENTARY DATA
