# Supplementary material for: RNA regulators responding to ribosomal protein S15 are frequent in sequence space
Source: Nucleic Acids Res. 2016 Aug 31;44(19):9331–41. doi: 10.1093/nar/gkw754 (PMC5100602; doi:10.1093/nar/gkw754)

Supplemental Material

Figure S1. SELEX Overview

(A) Diagram of *in vitro* selection process using Systematic Evolution of Ligands by EXponential Enrichment (SELEX). Our initial pool, “Mutant DNA Pool”, was based upon the Ec-mRNA 5’-UTR sequence, containing 30 randomized nucleotides as indicated, N_{30}. This pool was transcribed to generate the “Mutant RNA Pool”. This underwent a Negative Selection in the absence of Gk-S15 that removed RNA non-specifically interacting with nitrocellulose. Next, the pool underwent a selection in the presence of Gk-S15, “Positive Selection”. Gk-S15-binding RNAs were isolated from the protein, reverse transcribed, then PCR amplified using mutagenic PCR. This completed a round of selection. Periodically, sequences were isolated from a given round for sequencing, protein binding assays, or for cloning into our *in vivo* regulatory system. (B) Secondary structure diagram of the S15 responsive regulatory RNA from *E. coli* showing our randomized region.

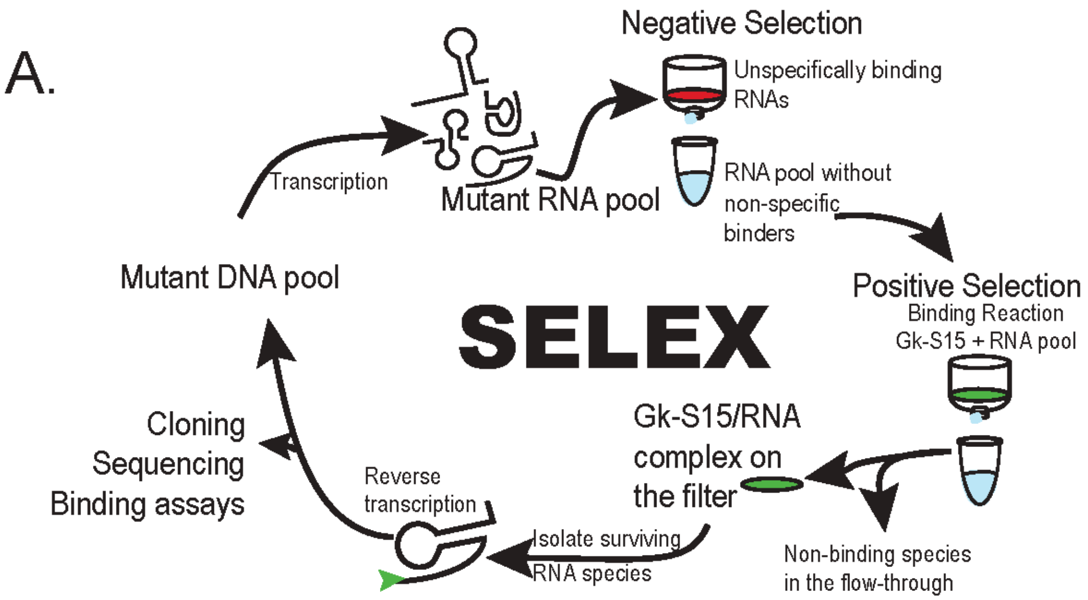

B. Randomized pool based on Ec-mRNA sequence

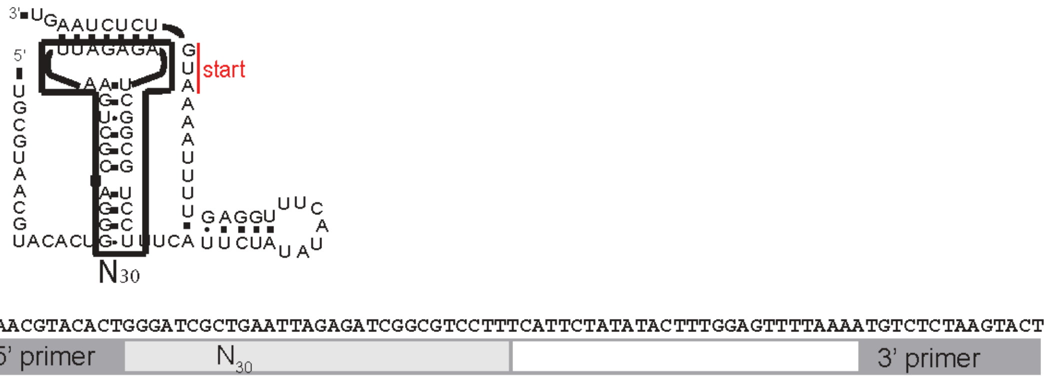

Figure S2. Pool Affinity for Gk-S15 in Rounds 0, 9, and 11.

(A) The protein concentration was decreased as indicated from round to round throughout the SELEX process. The affinity of the total RNA pool was assessed in rounds 0, 9, and 11 using nitrocellulose filter binding assays, ^{32}P-labeled RNA and serial dilutions of Gk-S15. (B) Binding curves for Round 0, 9, and 11 with Gk-S15. See Materials & Methods for calculation of K_D and fraction bound.

A.

Round	Gk-S15 (nM)	K_D (nM)
0	0	1329
1	1250	-
2	1250	-
3	1000	-
4	1000	-
5	700	-
6	250	-
7	250	-
8	250	-
9	250	253
10	250	-
11	100	150

B.

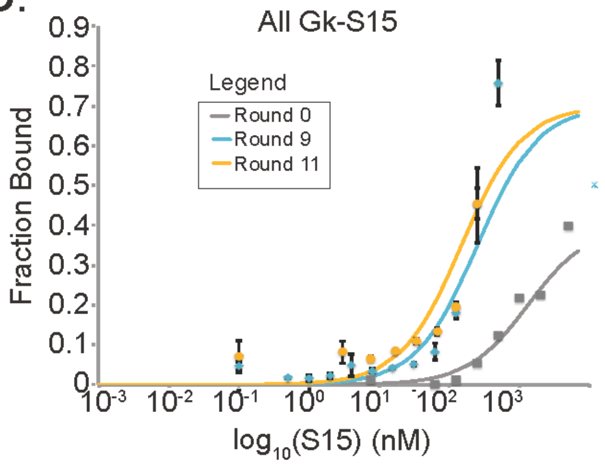

Figure S3. Table of Primers and Reference Sequences

(A) To amplify SELEX population (B) For cloning and sequences from pCR.2.1 TOPO vector (C) Site-directed mutagenesis of RNA 11-1 (D) Site-directed mutagenesis of RNA 11-2 (E) For cloning synthetic RNAs into pBS3 vector (F) For construction of pS15 vector (G) Site-directed mutagenesis of pGk-S15

A. To amplify SELEX population

Name	Sequence
361	5'-TAATACGACTCACTATAGGTGCGTAACGTACACT
509	5'-AGCTGTTGCTTCAGTACTTAGAGACATT

B. For cloning and sequencing from pCR.2.1 TOPO vector

Name	Sequence
34	5'-GTTTTCCCAGTCACGACGTTGTA
35	5'-CAGGAAACAGCTATGAC

C. RNA 11-1 site directed mutagenesis

Name	Sequence
836-M1F	5'-CAAGTAATACGACTCACTATAGGACACTTCCTTCGCTTATTCGGAGTAG
1398-m2F	5'-TAATACGACTCACTATAGGTtgGTAACGTACACT
1399-M3F	5'-TAATACGACTCACTATAGGTtgGTAACcgACACT
861-M4R	5'-CTCCAAAGCATACAGAATGATCACG
862-M5R	5'-GCATACAGAATGATCACGTGATCTAC
1105-M6F	5'-GCTTATTCG GAG TAA TCT GTA TGC TTTGGAG
1106-M6R	5'-CTCCAAAGCATACAGATTACTCCGAATAAGC
1252-M7F	5'-TAATACGACTCACTATAGGTGCGTAACGTACAGATCCTTCG
1253-M8R	5'-AGTACTTAGAGACATTTTAAAAGACCAAAGC
11-1-M9	1253R ON M7 TEMPLATE
11-1-M10	1252 ON M11 TEMPLATE
1465-M11F	5'-CGCTTATTCGGTCTAGATCACG
1466-M11R	5'-CGTGATCTAGACCGAATAAGCG
859-M12R	5'-TTAGAGACATTTTAAAACTCCAAAGCATACAG

860-M13R	5'-GACATTTTAAAACTCCAAAGCATACAGAATG
1083-m14F	5'-tgcgtaacgtacacttctagatcacgtgatcattctgtatgctttggagtttt
1084-m15F	5'-tgcgtaacgtacacttccttcgcttattcggagtagatcacttggagtttt
1101-M16F	5'-GAG TAG ATC GGG AGA TCA TTC TGT ATG
1102-M16R	5'-CATACAGAATGATCTCCCGATCTACTC
1103-M17F	5'-CTT ATT CGG AGT AGC ATT CTG TAT GC
1104-M17R	5'-GCATACAGAATGCTACTCCGAATAAG
1250-M18F	5'-TAATACGACTCACTATAGGTGCGTAACGCACACT
11-1-M19	860R ON M6 TEMPLATE

D. RNA 11-2 site directed mutagenesis

Name	Sequence
1463-11-2-M1F	5'-AATAGATCATTCGGGATACTGTGGAGC
1464-11-2-M1R	5'-GCTCCACAGTATCCCGAATGATCTATT

E. To clone selected sequences in vivo:

Name	Sequence
673F	5'-caagaattcTGCGTAACGTACACT
52R	5'-ACGCGTCGACAGCTGTTGCTTCAGTACTTAGAGACA

F. To clone pS15

Plasmid	Primer	Primer Sequence	Reference Sequence
pEc-S15	411F	5'-CACGAGCTCAGGAGGTTTTAAAATGTCTCTAAGTACTGAAGCACAG	AtgtctctaagtactgaagcaacagctAaaatcgtttctgagtttggtcgtgacgcaaacgacaccggttctaccgaagttcaggtagcactgctgactgcacagatcaaccacctgcagggccactttgcagagcacaaaaaagatcaccacagccgtcgtggtctgctgcgcatggtttctcagcgtcgtaaactgctcgactacctgaaacgtaaagacgtagcacgttacacccagctcatcgagcgcctgggtctgcgtcgctaa
	18R	5'-GCTCTAGATTAGCGACGCAGACCCAGGCGC	
pTt-S15	1372F	5'-CACGAGCTCAGGAGGTTTTAAAAtgcccatcacgaaggaagag	AtgcccatcacgaaggaagagaagCagaaggtcatccaggagttcgcccCgcttccccggggacacggggagcAccgaggtgcaggtggcgctccttAccctgaggatcaaccggctt

	565R	5’-cacgagctcggagggaaaacatgcccatcacgaaggaagag	tccGagcacctcaaggtccacaagaagGaccaccactcccaccgcggcctcCtgatgatggtgggccagcgccgcAggctcctccgctacctccagcggGaggaccccgagcggtaccgggccCttattgagaagctgggcatccggggttaa
pGk-S15	1371F	5’-CACGAGCTCAGGAGGTTTTAAAAtggcattgacgcaggagcgC	AtggcattgacgcaggagcgcaaaCgcgaaatcatcgagcagtttaaaAtccatgagaacgacactggttctCcggaagtgcaagttgcgatcctgAcggagcaaatcaacaacttgaacGagcatttgcgcattcataaaaaaGaccatcattcacggcgcggcttgCtgaaaatggtcgggaagcgccgcAacttattggcctacttgcgcaagAaagatgtggcgcgctaccgtgaaTtgattgagaaacttggattacgtcgataa
	23R	5’-GCTCTAGATTATCGACGTAATCCAAGTTTCTCAATC	

G. pGk-S15 site-directed mutagenesis

Name	Sequence
789-GKrpsOE40L-F	5’-GAGCAAATCAACAACTTGAACCTGCATTTGCGCATTC
790-Gk-rpsOE40L-R	5’-GAATGCGCAAATGCAGGTTCAAGTTGTTGATTTGCTC
791-GKrpsOD48L-F	5’-gcgcattcataaaaaactccatcattcacggcgcggc
792-GKrpsOD48L-R	5’-gccgcgccgtgaatgatggagtttttatgaatgcgc
793-GKrpsOY68A-F	5’-cgcaacttattggccgccttgcgcaagaaagatgtg
794-GkrpsOY68A-R	5’-cacatctttcttgcgcaaggcggccaataagttgcg
795-GkrpsOE79L-F	5’-GTGGCGCGCTACCGTCGTCTTTGATTGAGAAACT
796-GkrpsoE79L-R	5’-AGTTTCTCAATCAAAGACGACGGTAGCGCGCCAC

Figure S4. Individual RNAs Isolated from Round 11 Have Diverse Sequence and Predicted Structure

(Top) Six individual RNAs were isolated from the Round 11, and their sequence aligned using MultAlin (Corpet 1988), high consensus (black, e.g. the 5' and 3' primers), low consensus (red), and no consensus (pink). The primer regions and the N30 region are indicated. (Bottom) The predicted structure for all sequences was analysed using RNAfold of the Vienna RNA Package (Lorenz 2011).

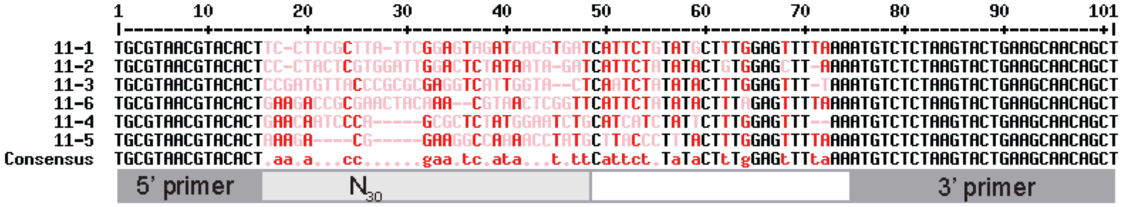

>11-1

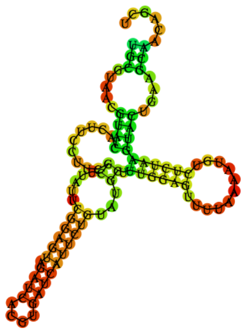

>11-2

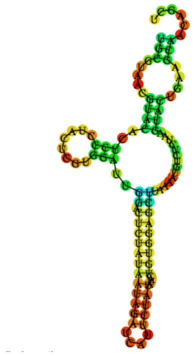

>11-3

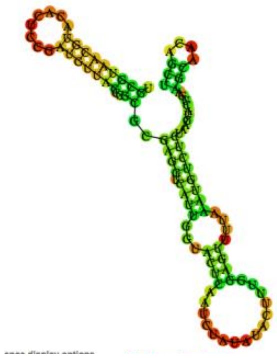

>11-4

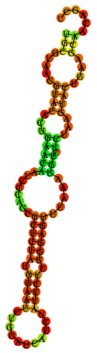

>11-5

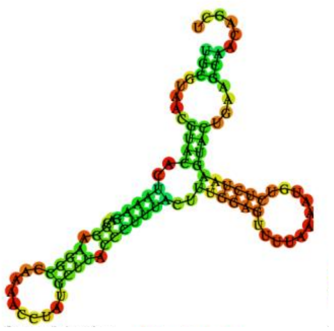

>11-6

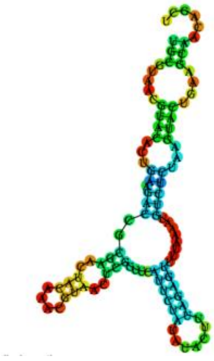

Figure S5. RNAs Isolated from Round 11 Bind Gk-S15

The binding affinity for the individual RNAs isolated from the Round 11 pool was assessed using nitrocellulose filter binding assays, ^{32}P-labeled RNA, and serial dilutions of Gk-S15. Shown is the individual binding curves for Gk-mRNA (black), Ec-mRNA (blue), 11-1 (pink), 11-2 (orange), 11-3 (red), 11-4 (yellow), 11-5 (green), and 11-6 (purple), which were used to calculate the K_D and F_{MAX} for each individual RNA (Table 1). Lines represent calculated best fit to the equation $Fb=(F_{max}*[S15])/([S15]+K_D)$ that represents a 1:1 binding interaction between RNA and protein. Most of the RNAs display a good fit to this equation, but RNA 11-5 shows a relatively poor fit and may not be a 1:1 interaction.

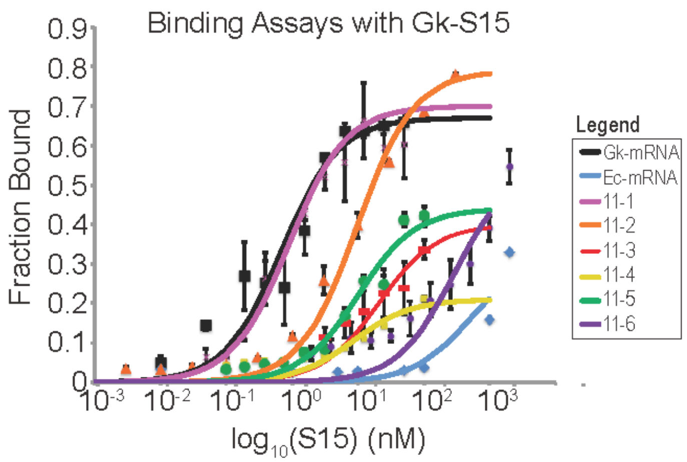

Figure S6. Miller Units + and - L-arabinose

Miller Units + and – L-arabinose from the *in vivo* regulation assays that were used to calculate fold-repression values. For all, solid bars indicate + L-arabinose conditions, striped bars indicate –L-arabinose conditions. (A) pGk-S15 and pEMTPY interactions with Gk-mRNA, Ec-mRNA, RNA 11-1, and RNA 11-2, dark gray bars are pGk-S15 and white/light gray bars are pEMTPY (B) pGk-S15 and pEMTPY interactions with RNA 11-3, RNA 11-4, RNA 11-5 , and RNA 11-6, dark gray bars are pGk-S15 and white/light gray bars are pEMTPY (C) pTt-S15 and pEc-S15 interactions with RNA 11-1, RNA 11-4, and RNA 11-6, light gray bars are pTt-S15, dark gray bars are pEc-S15 (D) RNA 11-1 interactions with pGk-S15-E40L, pGk-S15-D48L, pGk-S15-Y68A, and pGk-S15-E79L.

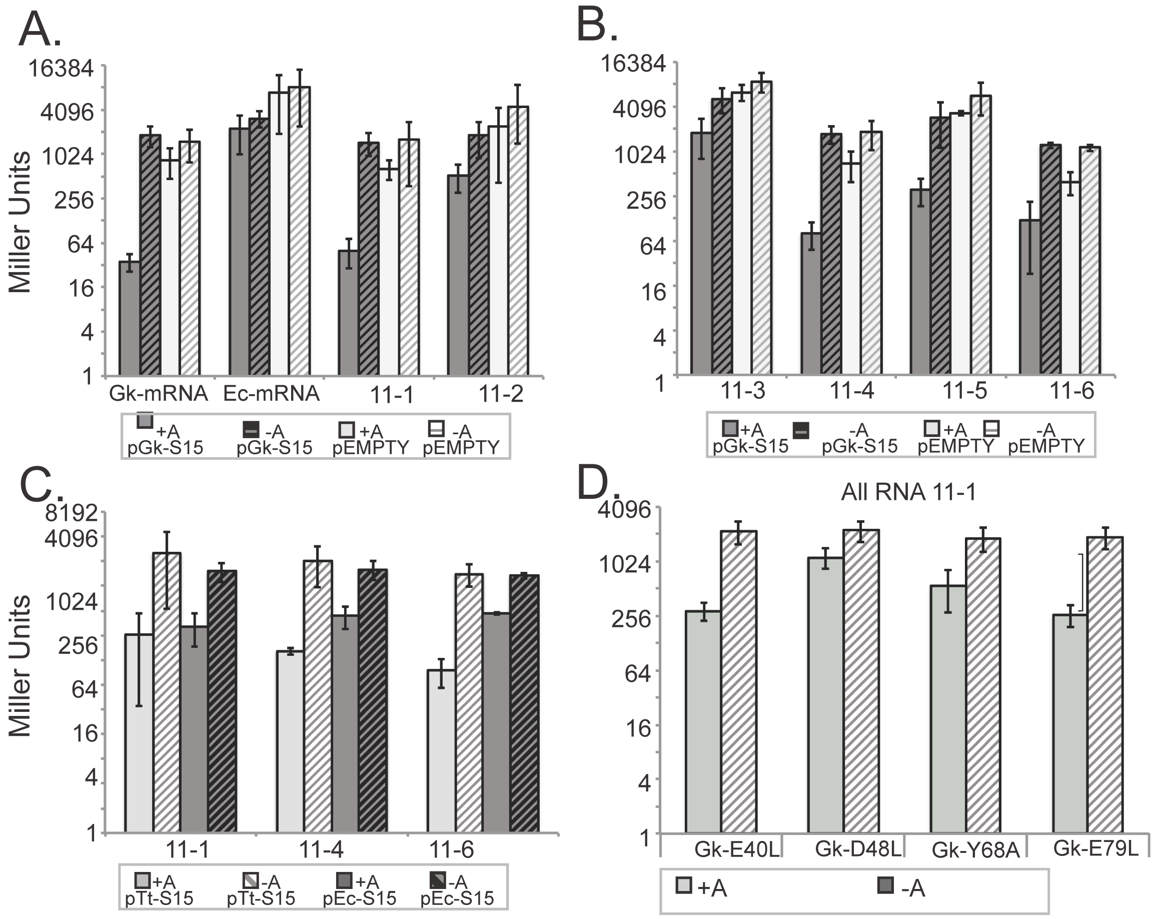

Figure S7. RNAs 11-1 and 11-2 Bind the S15 Homolog from *T. thermophilus*

The binding affinity for the RNAs 11-1 and 11-2 was assessed using nitrocellulose filter binding assays, ^{32}P-labeled RNA, and serial dilutions of S15 purified from either *T. thermophilus* (Tt-S15) or *E. coli* (Ec-S15). Shown are the individual binding curves, which were used to calculate the K_D and F_{MAX} for each individual RNA. Data corresponding to Gk-S15 is re-plotted from Figure S4 for comparison. See Materials & Methods for additional information on calculations used to find K_D and F_{MAX}.

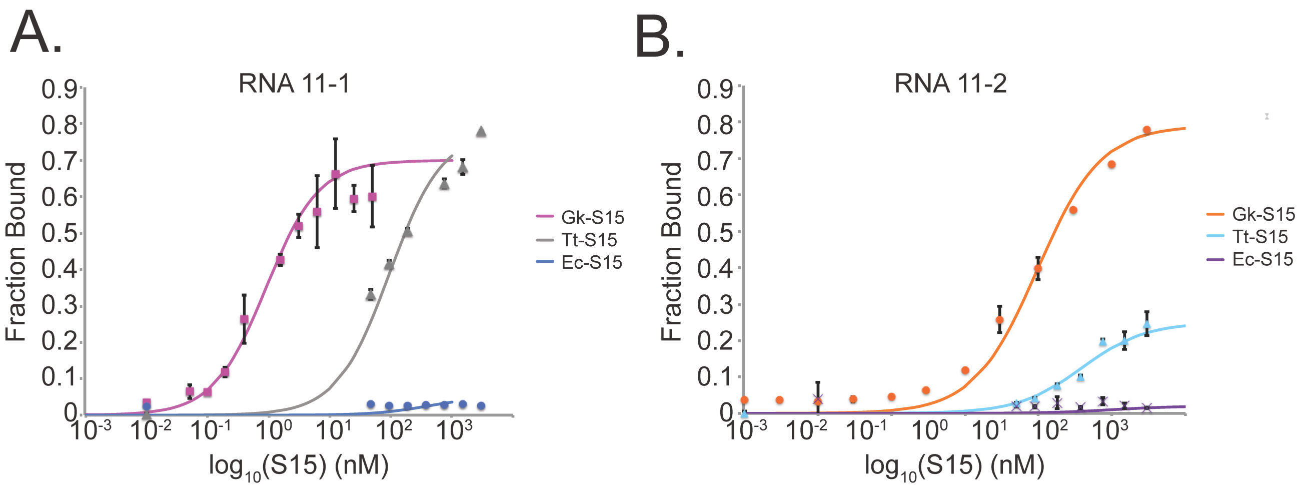

Figure S8. All RNA 11-1 Mutant Binding Assays with Gk-S15

Table of values for *in vitro* binding assays for all RNA 11-1 mutants with Gk-S15. See Figure S9 for binding curves.

RNA	K_D (nM)	STE	F_{MAX}	STE	Notes
11-1	0.9	2.0E-02	0.70	2.1E-03	
11-1-M1	>100	n/a	n/a	n/a	Del 5’ end – U10
11-1-M2	56.5	5.74	0.56	2.0E-02	G2-C3 →U2-G3
11-1-M3	20.9	6.3	0.85	3.7E-03	11-1-M2 + G9-U10 →C9-G10
11-1-M4	7.7	5.89	0.36	5.8E-03	Del U66 – 3’ end
11-1-M5	32	34	0.064	4.3E-02	Del U59 – 3’ end
11-1-M6	4.63	0.8	0.69	2.3E-02	Del G33-U49 → Ins GUAA
11-1-M7	>300	n/a	n/a	n/a	C14-U15 → G14-A15
11-1-M8	84.5	14.5	0.71	1.4E-02	A64-G65 → U64-C65
11-1-M9	>300	n/a	n/a	n/a	M7 + M8
11-1-M10	>300	n/a	n/a	n/a	M7 + M11
11-1-M11	>300	n/a	n/a	n/a	A32-G33 → U32-C33
11-1-M12	0.6	8.6E-02	0.42	1.0E-02	Del G83 – 3’ end
11-1-M13	0.81	2.5E-01	0.52	1.5E-02	Del U78 – 3’ end
11-1-M14	>300	n/a	n/a	n/a	Del C14-U28
11-1-M15	>300	n/a	n/a	n/a	Del G42-U59
11-1-M16	4.74	2.3	0.77	1.0E-02	A40-U43 → GGGA
11-1-M17	3.94	0.50	0.59	4.0E-02	A37-U46 → del
11-1-M18	10.9	2.7	0.61	3.0E-02	U10 → C10
11-1-M19	68.8	20.1	0.69	1.7E-02	M13 + M6

Figure S9. All RNA 11-1 Mutagenesis Filter Binding Assay Results

(A) Filter-binding curves for 11-1 mutants M1-M5, (B) filter binding curves for 11-1 mutants M6-M10, (C) filter-binding curves for 11-1 mutants M11-M15 (D) filter binding curves for 11-1 mutants M16-M19.

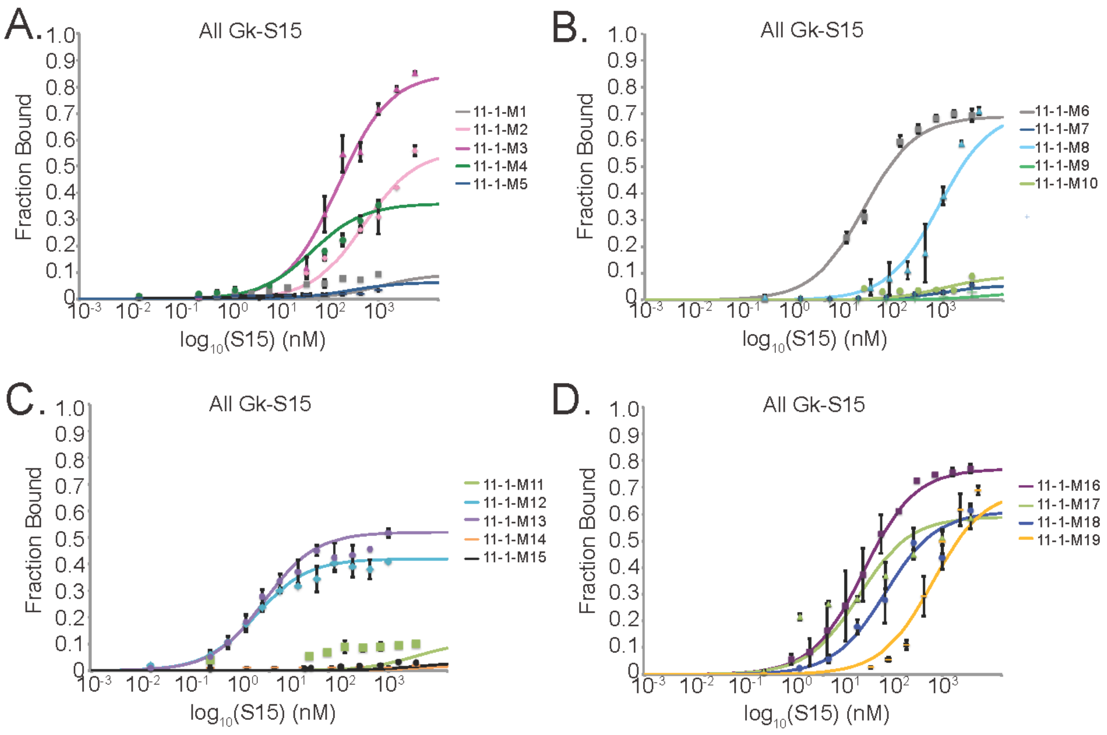

Figure S10. *In vitro* Mutagenesis Assays with Gk-S15 Plotted to an Alternative Potential Secondary Structure for RNA 11-1

Truncation sites and specific mutations to 11-1 are shown with an arrow. The Shine Dalgarno sequence is bolded, a bar is placed over the AUG start codon.

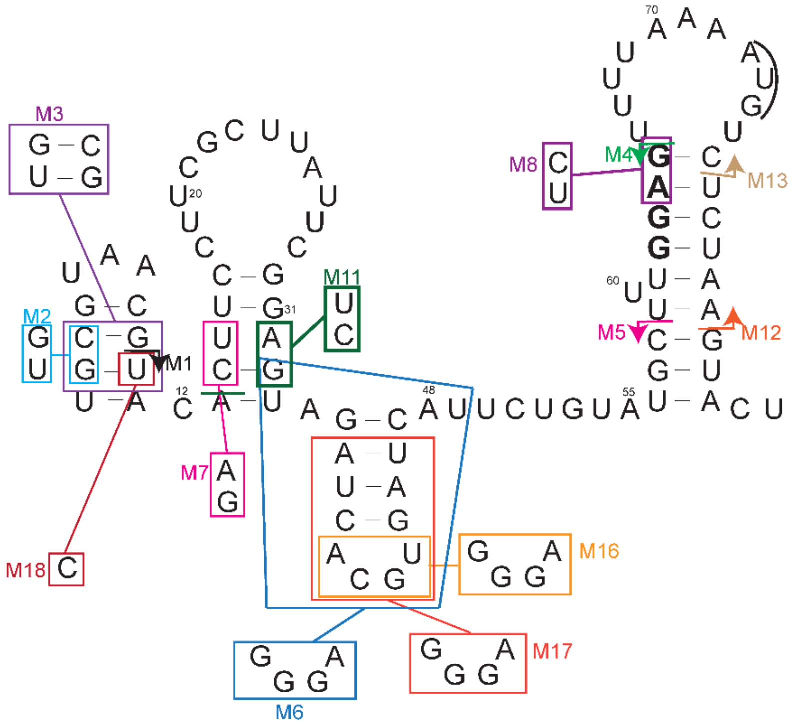

M1: del 5’ end - U10
M4: del U66 - 3’ end
M5: del U59 - 3’ end
M9: M7 + M8
M10: M7 + M11
M12: del G83 - 3’ end
M13: del U78 - 3’ end
M14: del C14 - U28
M15: del G42 - U59
M19: M13 + M6

Figure S11. Footprinting Data Mapped to the Alternative Structure for RNA 11-1

In-line (IL), RNase A (A), RNase VI (VI), and lead(II)-probing (Pb) cleavage products are indicated.

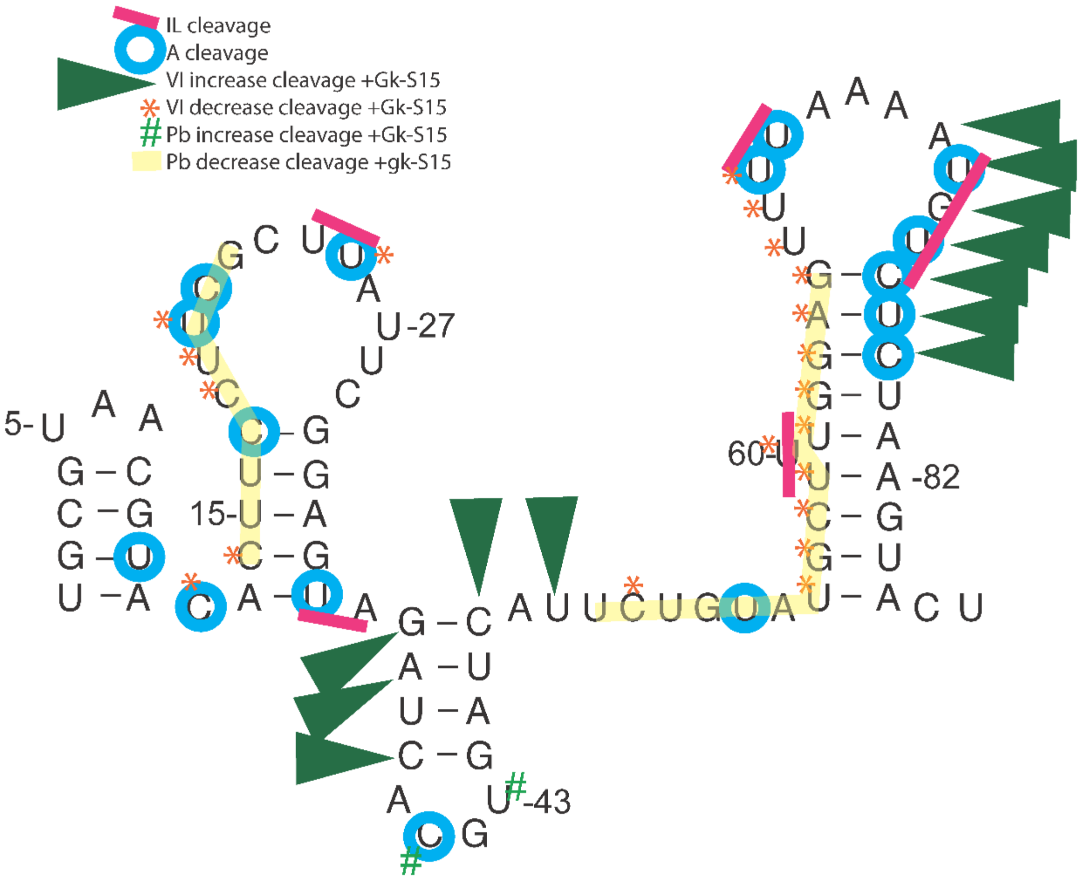

Figure S12. Structural probing for 11-2 and binding curves for Gk-S15 binding assays with RNA 11-2-M1

Structure probing assays show the protein-binding site on 11-2. Nucleotide numbering is based on common numbering in Figure 5. For all individual gels, no reaction (N), hydroxyl cleavage (OH), and denaturing RNase T1 ($T1^{SEQ}$), all cleavage products have been separated by denaturing 10% PAGE. (**A**) RNase T1 (T1), RNase A (A), and RNase VI (VI) cleavage products in the absence of Gk-S15. (**B**) Two independent replicates of in-line probing reactions (IL). (**C**) RNase VI (V1), RNase (A) in the presence and absence of 200 nM Gk-S15. (**D**) Binding curve of mutation 11-2-M1, demonstrating that the lead(II) protection sites are likely involved in protein binding. Data for RNA 11-2 is re-plotted from Figure S5 for comparison.

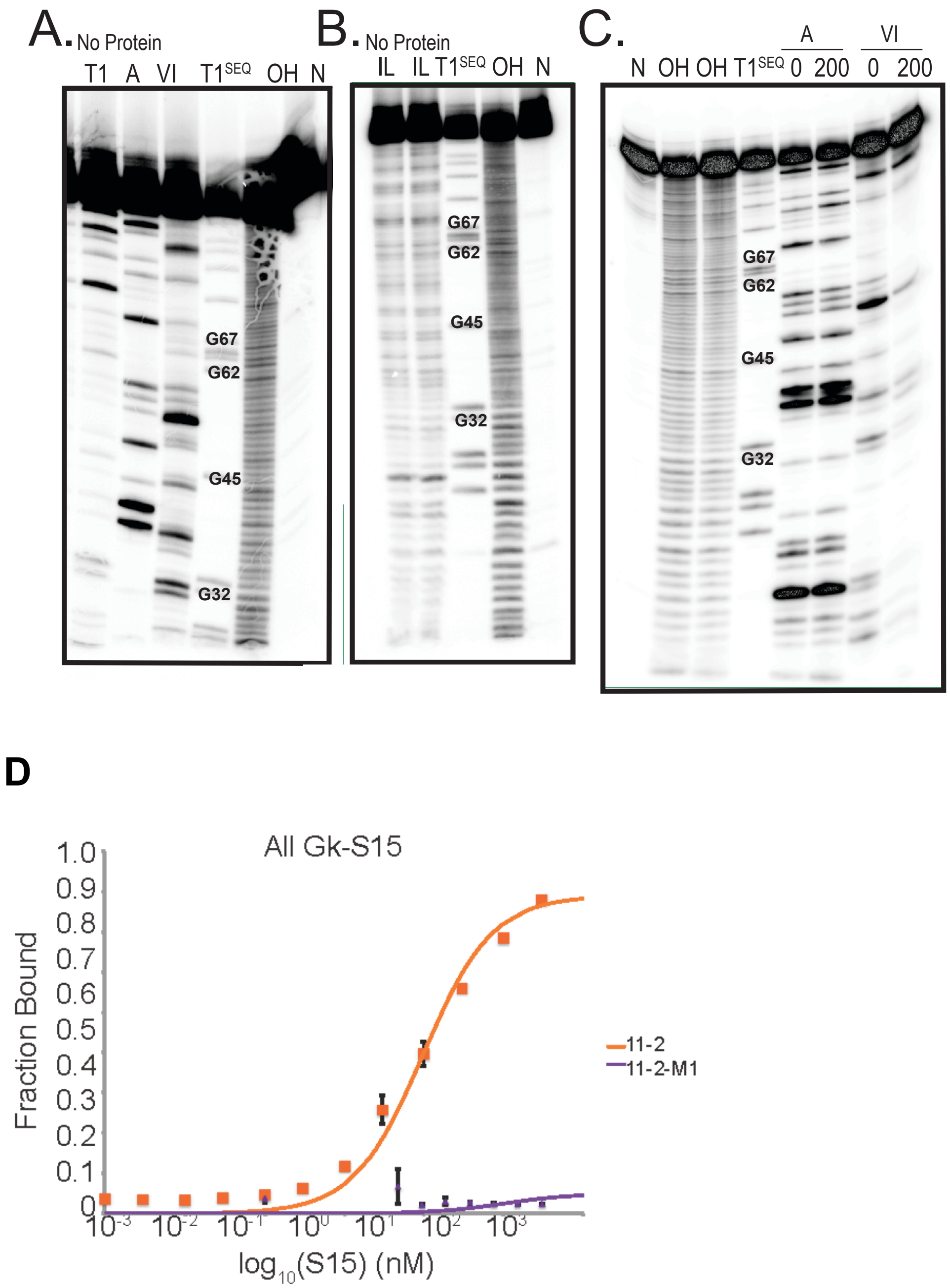

Supplement: SUPPLEMENTARY DATA [file supp_gkw754_nar-01789-z-2016-File007.pdf]
